# Design, Synthesis, and Characterization of Novel Coordination Compounds of Benzimidazole Derivatives with Cadmium

**DOI:** 10.3390/pharmaceutics14081626

**Published:** 2022-08-03

**Authors:** Anita Raducka, Marcin Świątkowski, Izabela Korona-Głowniak, Barbara Kaproń, Tomasz Plech, Małgorzata Szczesio, Katarzyna Gobis, Agnieszka Czylkowska

**Affiliations:** 1 Institute of General and Ecological Chemistry, Faculty of Chemistry, Lodz University of Technology, Żeromskiego 116, 90-924 Łódź, Poland; marcin.swiatkowski@p.lodz.pl (M.Ś.); malgorzata.szczesio@p.lodz.pl (M.S.); 2 Department of Pharmaceutical Microbiology, Medical University of Lublin, Chodźki 1, 20-093 Lublin, Poland; iza.glowniak@umlub.pl; 3 Department of Clinical Genetics, Medical University of Lublin, Radziwiłłowska 11, 20-080 Lublin, Poland; barbara.kapron@umlub.pl; 4 Department of Pharmacology, Medical University of Lublin, Radziwiłłowska 11, 20-080 Lublin, Poland; tomasz.plech@umlub.pl; 5 Department of Organic Chemistry, Faculty of Pharmacy, Medical University of Gdansk, Gen. Hallera 107, 80-416 Gdańsk, Poland; katarzyna.gobis@gumed.edu.pl

**Keywords:** metal complexes, antitumor, antiviral, metal-based drugs, medicinal chemistry, biologically active compounds, drug discovery, pharmacological activity, lung cancer, glioblastoma, neuroblastoma, adenocarcinoma, cadmium (II) coordination compounds, benzimidazole derivatives, thermogravimetric analysis, FTIR spectra, ADME analysis, MTT assay, antimicrobial activity

## Abstract

Four complexes of Cd(II) with benzimidazole derivatives were synthesized and named C1, C2, C3, and C4. All coordination compounds were characterized through elemental analysis (EA), flame atomic absorption spectrometry (FAAS), Fourier-transform infrared spectroscopy (FTIR), thermogravimetric analysis coupled with mass spectrometry) (TG-MS), a cytotoxicity assay (MTT (3-(4,5-dimethylthiazol-2-yl)-2,5-diphenyltetrazoliumbromide)), and computational chemical analysis for absorption, distribution, metabolism, and excretion (ADME). All of the obtained results are compatible and are consistent with the respective structures of the obtained compounds and their properties. The various techniques used allowed the determination of the composition, proposed structure of the compounds, their thermal stability and thermal properties, and the method of coordination between the metal (II) ion and the ligand. The ADME technique was also used to estimate the physicochemical and biological properties. The antitumor activity of the compounds was determined with an MTT assay on the glioblastoma (T98G), neuroblastoma (SK-N-AS), and lung adenocarcinoma (A549) cell lines, as well as normal human skin fibroblasts (CCD-1059Sk). Compound C2 was found to have potential antitumor properties and to be effective in inhibiting the growth of neuroblastoma cells. The antimicrobial activity of Cd complexes, free ligands, and reference drugs was tested against six strains of Gram-positive bacteria, five strains of Gram-negative rods, and three strains of yeasts. Compound C3 significantly increased activity against Gram-positive bacteria in comparison to the ligand.

## 1. Introduction

The creative design of new, functional coordination compounds plays a crucial role in the field of medical chemistry. An important feature of the new substances is their potential use as pharmacological agents. N-heterocyclic ligands deserve attention due to their coordination abilities and selected pharmaceutic properties. Particular attention should be paid to targeted anticancer chemotherapy. An important group of anticancer drugs are the coordination compounds of platinum. Their precursor and, at the same time, main representative is cisplatin. The chemotherapeutic potential of cisplatin in the treatment of cancer was first described in 1969 [1]. Currently, it is used in mono- and multi-drug therapies for the treatment of many cancers, especially testicular and ovarian cancer, head and neck cancer, and alveolar or small cell lung cancer [2,3,4,5,6,7,8,9]. Unfortunately, due to the toxic side effects (neuro- and nephrotoxicity) [10,11,12,13], narrow spectrum of action, and innate or acquired resistance of neoplastic cells to compounds of platinum [14,15,16], their use is limited, which is why research is carried out on chemotherapeutic agents that are based on cadmium ions together with selected organic ligands. Despite the fact that cadmium is one of the most toxic heavy metals and is in the first group of carcinogens according to the International Agency for Research on Cancer (IARC), its participation in complex compounds with anticancer activity should be considered [17,18,19]. The results of research on the cytotoxic properties of cadmium complexes showed that these are compounds with a significant degree of toxicity not only towards cancerous cells, but also towards normal cells; therefore, in their therapeutic effects, they resemble cisplatin [20,21,22]. Research on new cadmium complexes offers hope for the emergence of new and highly effective anticancer drugs, with a wide spectrum of action and a beneficial pharmacological profile. It is well known that compounds with anticancer activity that are based on endogenous metals, such as Cu and Cd, are less toxic than platinum complexes. Copper-containing coordination compounds have also been shown to be promising anticancer agents. Their activity takes various mechanisms into account, e.g., the inhibition of proteasome activity, reactive oxygen formation, telomerase activity, paraptosis, DNA intercalation, and DNA degradation [23]. This study was designed to explore the role of new derivatives as potential anticancer agents against glioblastoma (T98G), neuroblastoma (SK-N-AS), and lung adenocarcinoma (A549) cell lines, as well as normal human skin fibroblasts (CCD-1059Sk). This study confirmed that the conversion of ligands into the respective metal complexes significantly improved their anticancer properties. All of them showed much stronger activity than etoposide, which is a component of the therapy for glioblastoma, neuroblastoma, and lung cancer. There are many publications in the literature on the physicochemical and biological properties of metal coordination compounds with various derivatives of benzimidazole [24,25,26,27,28]. This fact proves that this is an interesting subject of research.

## 2. Materials and Methods

### 2.1. Materials and Analysis

The substrates used for ligand synthesis, as well as CdCl_2_·2H_2_O, were purchased from Sigma Aldrich (Warszawa, Poland). Glioblastoma (T98G), neuroblastoma (SK-N-AS), and lung adenocarcinoma (A549) cell lines and normal human skin fibroblasts (CCD-1059Sk) were obtained from the American Type Culture Collection (Manassas, VA, USA). High-glucose DMEM, DMEM/F12, MEM, fetal bovine serum, penicillin, and streptomycin were all obtained from Sigma-Aldrich.

### 2.2. Methods and Instruments

In order to compare the results of our work on coordination compounds with benzimidazole derivatives, the same techniques and methods were used.

Samples of complexes (about 20 mg) were digested in a concentrated mixture of 36% HCl (1 mL) and 65% HNO_3_ (6 mL). The contents of Cd (II) in the solid complexes were determined with an F-AAS spectrometer (Analytik Jena, contraAA 300, Jena, Germany) with a continuous source of light and using an air/acetylene flame (Analytik Jena, contraAA 300). Absorbances were measured at the analytical spectral lines at 228.8 nm for Cd (II). The limit of quantification was 0.004 mg/L for Cd (II). The solid samples were decomposed using an Anton Paar Multiwave 3000 (Graz, Austria) closed-system instrument. Mineralization was carried out for 45 min at 240 °C under a pressure of 60 bar. The contents of carbon, hydrogen, and nitrogen were determined with an instrument from Vario Micro Company Elementar Analysensysteme GmbH (Langenselbold, Germany). FTIR spectra were recorded with an IR Tracer-100 Schimadzu Spectrometer (4000–600 cm^−1^ with an accuracy of recording of 1 cm^−1^, Kyoto, Japan) using KBr pellets. The thermal analyses were carried out with a Netzsch STA 449 F1 Jupiter thermoanalyzer (Netzsch-Geratebau GmbH, Selb, Germany) coupled with a Netzsch Aeolos Quadro QMS 403 mass spectrometer (Netzsch-Geratebau GmbH, Selb, Germany). The samples were heated up to 1000 °C in corundum crucibles with a heating rate of 10 °C/min in an atmosphere of synthetic air (20% O_2_, 80% N_2_). The cytotoxic effects of the compounds were evaluated using glioblastoma (T98G), neuroblastoma (SK-N-AS), and lung adenocarcinoma (A549) cell lines and normal human skin fibroblasts (CCD-1059Sk). T98G and SK-N-AS cells were cultured in high-glucose DMEM and A549 cells were cultured in DMEM/F12, while CCD-1059Sk cells were cultured in MEM. All media were supplemented with 10% (FBS), 100 U/mL of penicillin, and 100 mg/mL of streptomycin (PenStrep). Cells were incubated at 37 °C in a humidified atmosphere of 5% CO_2_. Stock solutions were prepared by dissolving the compounds in sterile dimethyl sulfoxide (DMSO) to obtain a concentration of 50 mg/mL. The cells were seeded into sterile 96-well plates (Nunc) at a density of 1 × 10^5^ cells/mL. After 24 h of incubation, the medium was removed from each well, and then the cells were incubated for the next 24 h with different concentrations of the tested compounds (1–100 µg/mL) in their respective media containing 2% FBS. The cytotoxicity of the compounds was evaluated using an MTT assay [29], which was based on the conversion of 3-(4,5-dimethylthiazol-2-yl)-2,5-diphenyltetrazolium bromide (MTT) into dark-blue formazan crystals. Briefly, after 24 h of incubation of the cells with varying concentrations of the tested compounds, all culture media were removed from the plates. The cells were washed with PBS, and then 100 µL of medium containing 10% MTT solution (5 mg/mL) was added to each well. After 3 h of incubation, 100 µL (per well) of 10% SDS buffer solution was added to solubilize the formazan crystals. After overnight incubation, the absorbance was measured at 570 nm using a microplate reader (Epoch, BioTek Instruments). Experiments were repeated twice, and the measurements in each experiment were run in triplicate. The viability of the investigated cells was expressed as the percentage of the viability of the untreated cells, and the results were transformed into IC50 values (expressed as mean ± SD). The complexes were screened for antibacterial and antifungal activity by using the micro-dilution broth method with Mueller–Hinton broth for the growth of bacteria or Mueller–Hinton broth with 2% glucose for the growth of fungi [30]. The minimal inhibitory concentration (MIC) of the tested derivatives was evaluated for the panel of reference microorganisms from the American Type Culture Collection (ATCC), including Gram-negative bacteria (*Escherichia coli* ATCC 25922, *Salmonella* Typhimurium ATCC14028, *Klebsiella pneumoniae* ATCC 13883, *Pseudomonas aeruginosa* ATCC 9027, *Proteus mirabilis* ATCC 12453), Gram-positive bacteria (*Staphylococcus aureus* ATCC 25923, *Staphylococcus epidermidis* ATCC 12228, *Micrococcus luteus* ATCC 10240, *Enterococcus faecalis* ATCC 29212, *Bacillus subtilis* ATCC 6633, *Bacillus cereus* ATCC 10876), and fungi (*Candida albicans* ATCC 10231, *Candida parapsilosis* ATCC 22019, *C. glabrata* ATCC 90030). 

### 2.3. Statistical Analysis

The data were expressed as the mean ± SD values from three independent replicated experiments. P values of less than 0.05 in comparison with the control group according to Student’s *t*-test were considered to be statistically significant [31].

### 2.4. ADME Analysis

An ADME analysis was performed using the SwissADME service (Swiss Institute of Bioinformatics 2021) [32,33,34] and the ProTOX II service for the prediction of the toxicities of the tested compounds [35].

## 3. Results and Discussion

### 3.1. Synthesis

#### 3.1.1. Ligand Synthesis

**Scheme 1:**
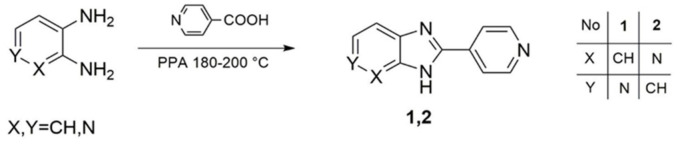
Synthesis of ligands 1 (**L1**) and 2 (**L2**).

**Scheme 2:**
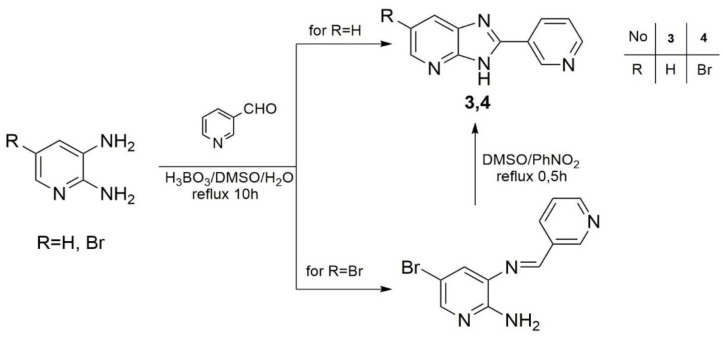
Synthesis of ligands 3 (**L3**) and 4 (**L4**).

Ligands **L1** and **L2** were synthesized through the condensation and cyclization of 2,3-diminopyridine or 3,4-diaminopyridine with isonicotinic acid in PPA (polyphosphoric acid) (Scheme 1) and ligands L3 and L4 were obtained by reacting 2,3-diaminopyridine or 5-bromo-2,3-diaminopyridine with 3-pyridinecarboxaldehyde in the presence of boric acid in a mixture of water and DMSO (Scheme 2). We previously described these syntheses [36].

#### 3.1.2. Complex Synthesis

**Scheme 3:**
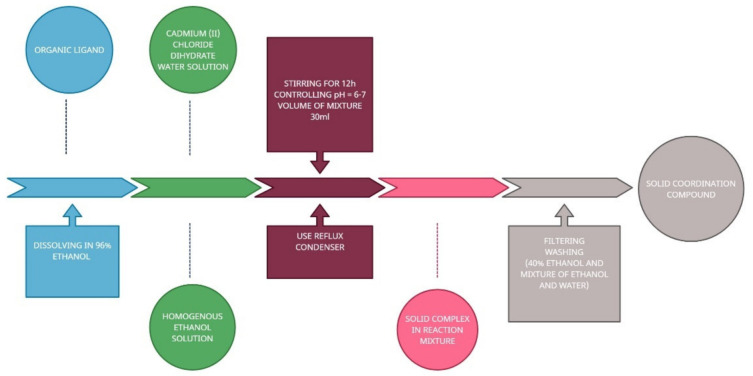
Synthesis of solid cadmium (II) coordination compounds.

The starting materials in the form of benzimidazole derivatives (0.25 mmol) and cadmium chloride dihydrates (0.25 mmol) used for the synthesis were dissolved in 96% *v/v* ethanol until homogeneous solutions were obtained. Then, they were mixed using a reflux condenser for about half a day. The total volume of the reaction mixture was 30 mL. The reaction was carried out under the conditions of a constant room temperature (25 °C) and controlled pH (6–7) until the formation of precipitates of the coordination compounds, which were then washed with 40% EtOH and a mixture of EtOH and H_2_O (vol = 1/1). The reaction products were air dried at room temperature (Scheme 3). The newly synthesized coordination compounds were characterized through an elemental C/H/N analysis, and the determination of Cd (II) content was carried out by using FTIR spectra and the TG-MS technique.

**C1**—**Cd(L1)Cl_2_∙H_2_O (C_11_H_10_N_4_OCdCl_2_)** (397.5403 g/mol), yield (43%), anal. calculated (%): Cd, 28.28; C, 33.23; H, 2,55; N, 14.09. Found (%): Cd, 28.57; C, 33.31; H, 2,39; N, 14.25.

**C2**—**Cd(L2)Cl_2_∙H_2_O (C_11_H_10_N_4_OCdCl_2_)** (397.5403 g/mol), yield (41%), anal. calculated (%): Cd, 28.28; C, 33.23; H, 2,55; N, 14.09. Found (%): Cd, 27.88; C, 33.45; H, 2.43; N, 14.15.

**C3**—**Cd(L3)Cl_2_∙2H_2_O (C_11_H_12_N_4_O_2_CdCl_2_)** (415.5556 g/mol), yield (38%), anal. calculated (%): Cd, 27,05; C, 31.79; H, 2.91 N, 13.48. Found (%): Cd, 27.21; C, 32.00; H, 2.59; N, 13.35.

**C4**—**Cd(L4)Cl_2_∙2H_2_O (C_11_H_11_N_4_O_2_CdBrCl_2_)** (494.4516 g/mol), yield (42%), anal. calculated (%): Cd, 22.73; C, 26.72; H, 2.24; N, 11.33. Found (%): Cd, 22.33; C, 26.37; H, 2.31; N, 11.19.

### 3.2. FTIR Spectra

**Figure 1:**
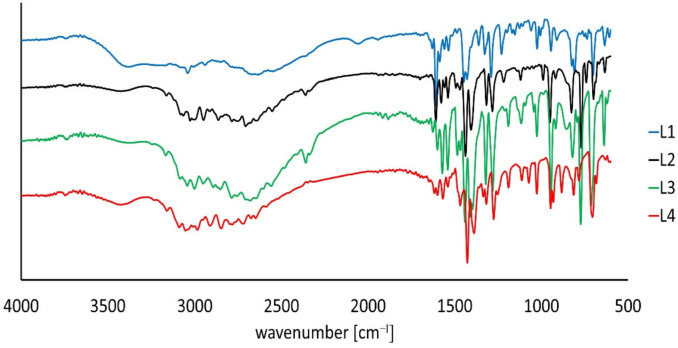
FTIR spectra of the free organic ligands.

**Figure 2:**
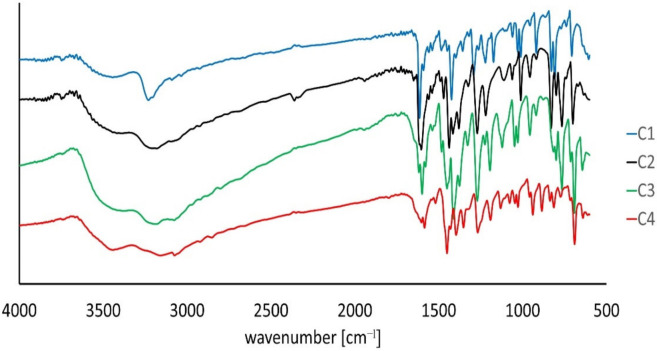
FTIR spectra of the cadmium (II) coordination compounds.

Figure 1 and Figure 2 show the spectra of the free ligands and the corresponding cadmium coordination compounds, respectively. During complexation, the vibrational modes of the free organic N-donors were changed. Comparing the spectra of each appropriate ligand and complex with each other, it can be seen that the fundamental ν(NH) from the imidazole ring, which was present in the organic ligands in the range of 3161–2591 cm^−1^ was shifted towards higher wavenumbers (3547–2928 cm^−1^) in coordination compounds **C2** and **C4**, thus showing that the NH group did not participate in binding. However, in the case of compounds **C1** and **C3**, we can say that these bands did not change because of a lack of coordination from the imidazole ring. In the spectra of the uncoordinated donors, the vibrations modes of ν(C=N) and ν(C=C) were visible in the ranges of 1627–1600 and 1483–1390 cm^−1^, respectively. As a result of the coordination between the metal ion and the ligand, these frequencies shifted towards higher or lower frequencies with the following peaks: for **C1**, 1622 and 1423 cm^−1^; for **C2**, 1629, 1602, 1434, and 1414 cm^−1^; for **C3**, 1598, 1450, and 1406 cm^−1^; for **C4**, 1584, 1450, and 1395 cm^−1^. For compounds **C1** and **C3**, we could see hypsochromic effect on ligands **L1** and **L3**, respectively. This is evidence of a different way of coordination in these coordination compounds. For all complexes, moving towards the lower wavelengths, we could observe bands in ranges of 1300–1012 and 910–690 cm^−1^, corresponding to the β(CH) and γ(CH) modes, respectively. By analyzing the presented spectra, it could be concluded that, in the case of compounds **C1** and **C3**, the coordination took place through the nitrogen atom contained in the pyridine ring and due to the slight interaction of the metal (II) and hydrogen atom coming from the imidazole ring. As for compounds **C2** and **C4**, it was clearly seen that the N-donor ligands were monodentate and coordinated via the nitrogen from the imidazole ring.

### 3.3. Thermal Study

**Figure 3:**
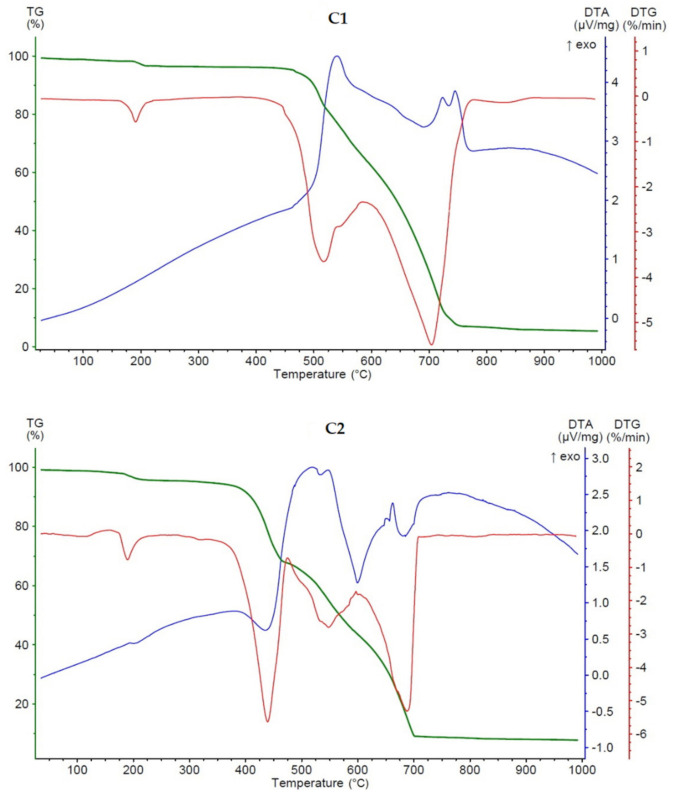

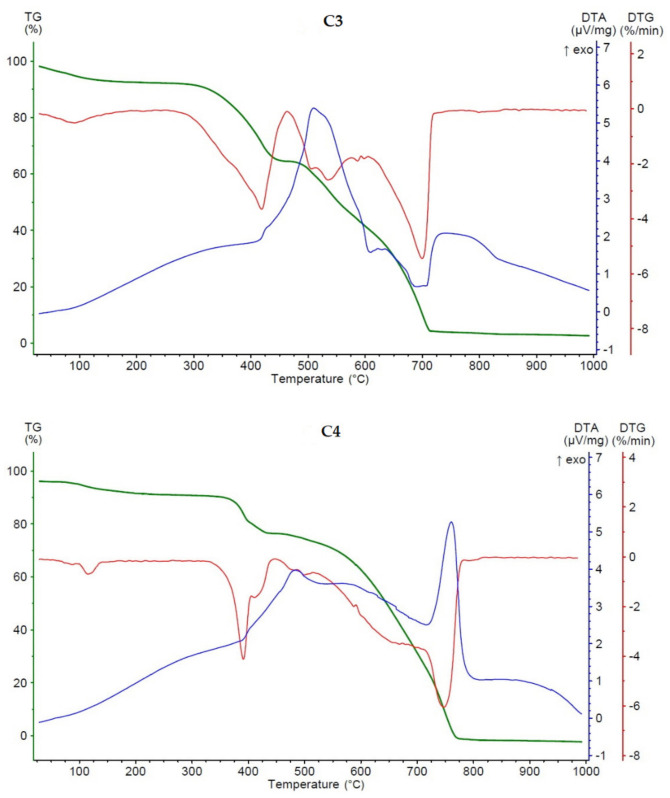
Thermal analyses of the cadmium (II) coordination compounds. TG (green), DTA (blue), and DTG (red) curves for complexes **C1**, **C2**, **C3**, and **C4**.

**Figure 4:**
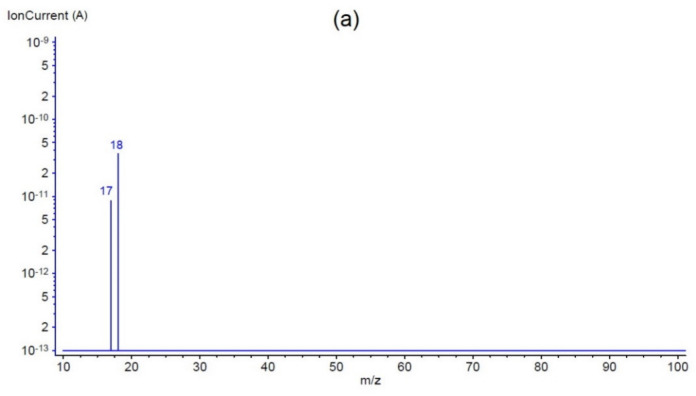

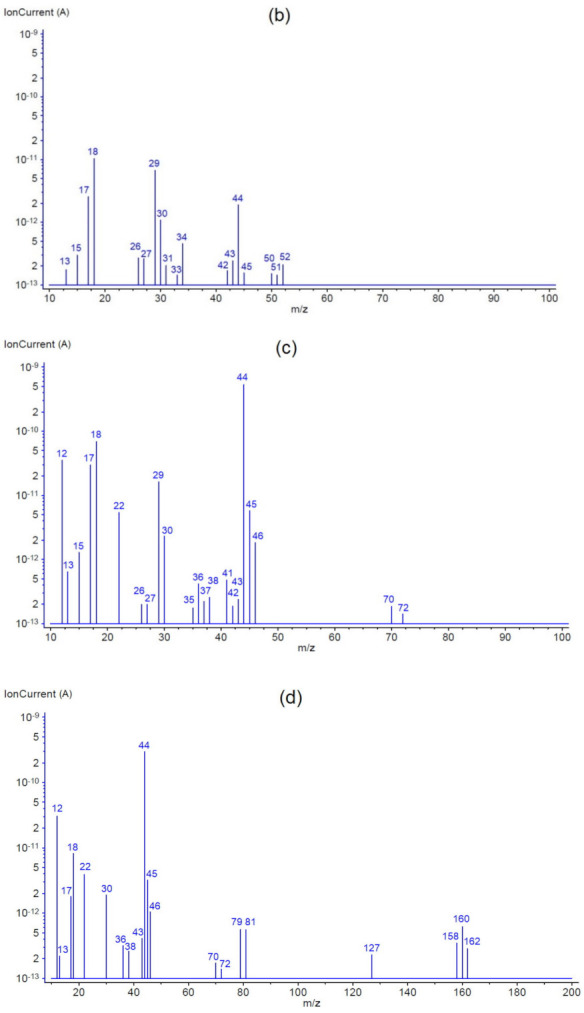
Representative mass spectra of volatile products of the first (**a**) and the second (**b**) stages of the thermal decomposition of **C1–C4**, as well of the third stage of the thermal decomposition of **C1–C3** (**c**) and **C4** (**d**). The correction of the mass spectra included subtracting the background spectrum and the application of automatic software correction for the carrier gas.

All studied compounds were hydrated; thus, the first mass loss registered on the TG curves corresponded to water removal (Figure 3 and Figure 4a). This process finished at temperatures in the range of 180–220 °C (Table 1), which could indicate that the water molecules formed coordination bonds with the cadmium cations [37]. The dehydrated compounds started to decompose at different temperatures (Table 1), which could be a consequence of their different structures. The location of the nitrogen atoms in the two pyridine rings of **C1** and **C2** favored the formation of polymeric compounds, whereas in **C3** and **C4**, it rather limited the propagation of the infinite structure. The thermal stability of coordination polymers is usually greater than that of discrete coordination compounds created from similar ligands [38]; hence, the structural diversity could be the most probable explanation for the greater thermal stability of **C1** and **C2** in comparison to that of **C3** and **C4**. The decomposition pathway of the organic ligand in the studied cadmium compounds was similar to that reported for analogous copper compounds [36]. However, the initial decomposition temperatures are generally higher for cadmium compounds. Firstly, the non-conjugated pyridine ring decomposed. The mass spectra at this stage for all of the studied compounds contained signals that were characteristic of pyridine fragmentation ions, i.e., HCN^+^ and C_4_H_4_^+^ [39], as well as of the combustion of heterocyclic organic moieties, i.e., H_2_O^+^, NO^+^, and CO_2_^+^ (Figure 4b). At temperatures in the range of 460–475 °C, the second step of organic ligand decomposition began (Table 1). For only **C1**, it was not possible to establish borders between the ligand decomposition substages due to their overlapping (Figure 3). The second substage of ligand decomposition was accompanied by the partial removal of the cadmium content in the form of cadmium chloride, a random amount of which evaporated above its melting point (564 °C) [40]. Therefore, the residual mass of cadmium oxide as a final product was lower than expected for **C1**–**C3** (Table 1). The calculated mass of cadmium oxide was above 30%. The presence of signals of Cl^+^ and Cl_2_^+^ ions in the mass spectra in this stage (Figure 4c) was evidence of the evaporation of CdCl_2_, which most likely underwent disintegration in the mass spectrometer. In the case of **C4**, total decomposition occurred. The entire cadmium content was removed in the form of both CdCl_2_ and CdBr_2_ (melting point: 568 °C) [40]. Bromide ions were generated during the decomposition of the imidazopyridine part of the ligand. Similarly to CdCl_2_, the signals of Br^+^ and Br_2_^+^ derived from CdBr_2_ were present in the mass spectra of the last decomposition stage of **C4** (Figure 4d). The total decomposition of cadmium coordination compounds containing bromine is a phenomenon that is well known in the literature [41].

### 3.4. MTT Cytotoxicity Assay

The ligands alone (**L1, L2, L3, L4**) and their Cd (II) metal complexes were screened for anticancer activity against the glioblastoma, neuroblastoma, and lung carcinoma cell lines (T98G, SK-N-AS, A549, respectively). Moreover, in order to assess the selectivity of the anticancer effect, the investigated compounds were also evaluated against normal human skin fibroblasts (CCD-1059Sk). Etoposide, which is a topoisomerase IIα inhibitor, was used as a reference drug. The results obtained confirmed that the conversion of the ligands into the respective metal complexes significantly improved their anticancer properties (Table 2). All of them showed much stronger activity than that of etoposide, which is a component of the therapy for glioblastoma, neuroblastoma, and lung cancer. Unfortunately, increased cytotoxicity of the metal complexes was also observed in relation to normal cells. Importantly, the neuroblastoma SK-N-AS cells, unlike T98G and A549 cells, exhibited greater sensitivity to the investigated cadmium complexes when compared to normal human cells. The most promising Cd (II) complex, i.e., **C2**, inhibited the growth of neuroblastoma cells five times more than etoposide, with an IC_50_ equal to 22.46 μM (vs. 115.25 μM for etoposide).

### 3.5. Antimicrobial Activity

The antimicrobial activity of the Cd complexes, free ligands, and reference drugs was tested against six strains of Gram-positive bacteria, five strains of Gram-negative rods, and three strains of yeasts. The antimicrobial properties were expressed as the MIC (minimum inhibitory concentration) in mg/L (Table 3). The antimicrobial activity of the Cd complexes was compared with the antimicrobial and antifungal properties of the appropriate ligands. Vancomycin (Van), ciprofloxacin (Cip), and nystatin (Nys) were used as the standard drugs. The tested ligands and complexes showed no bioactivity against Gram-negative bacteria, except for **C3**, which had significantly increased activity in comparison to its ligand. Against Gram-positive bacteria, moderate bioactivity was detected, with only a slight increase for staphylococci. Thus, the antibacterial efficiency of the tested complexes against Gram-positive bacteria decreased in the following order: **C1** > **C2** > **C4** > **C3**. Notably, all tested Cd complexes revealed good activity against yeasts in comparison to their ligands (MIC in the range of 7.8–125 mg/L).

### 3.6. ADME Analysis

**Figure 5:**
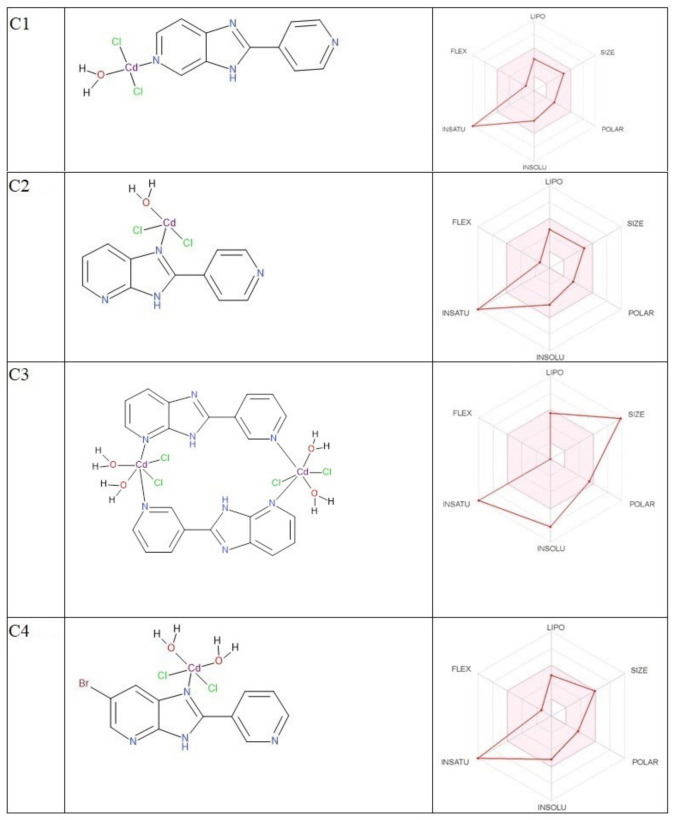
Bioavailability radars for the complexes.

**Figure 6:**
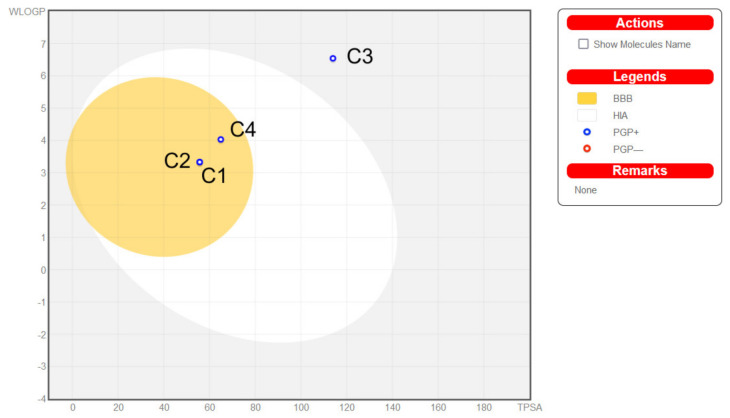
BOILED-Egg diagram for the complexes.

Bioavailability radars were used for all complexes (Figure 5). The pink-colored zone on the bioavailability radar was made in the SwissADME service; it represents the optimal range for each property to indicate the drug-likeness of a molecule: Lipophilicity (LIPO) was within the range –0.7 < XlogP3 < +5.0; molecular weight (SIZE) was within the range 150 g/mol < MW < 500 g/mol; polarity (POLAR) was within the range 20 Å^2^ < TPSA < 130 Å^2^; insolubility (INSOLU) was within the range 0 < logS < 6; insaturation (INSATU) was within the range 0.25 < Fraction Csp3 < 1; flexibility (FLEX) was within the range 0 < Num. rotatable bonds < 9. The non-dimer complexes met the rules of Lipinski [42], Ghose [43], Egan [44], Veber [45], and Muegge [46]. Only the dimer complex **C3** did not indicate good brain penetration or oral bioavailability. Coordination compound **C3** was too large, and its molecular weight exceeded the limit of 800 g/mol (831.11 g/mol) for being an obvious candidate as a drug. Gastrointestinal absorption and brain access are pharmacokinetically important properties when searching for a new drug. The BOILED-Egg graph was used to predict the penetration by computing the lipophilicity and polarity of the molecules. In the BOILED-Egg diagram (Figure 6), this complex clearly differed from the other coordination compounds. Servis ProTox II classified compounds **C1**, **C2**, and **C4** into toxicity class 4 (predicted LD50: 1000 mg/kg for **C1**; predicted LD50: 750 mg/kg for **C2**; predicted LD50: 450 mg/kg for **C4**) and compound **C3** into toxicity class 5 (predicted LD50: 2257 mg/kg).

## 4. Conclusions

Four complexes of Cd(II) with benzimidazole derivatives were successfully synthesized and named **C1**, **C2**, **C3**, and **C4**. The composition of the obtained complexes was confirmed by using FAAS, FTIR, and MS coupled with a TG study. All compounds were hydrates. After dehydration, non-conjugated pyridine ring degradation took place. The next step of ligand decomposition was accompanied by the partial removal of the cadmium content in the form of cadmium chloride, a random amount of which evaporated above its melting point. All of the results obtained from the thermogravimetric analysis and mass spectrometry were compatible with the respective structures of the obtained compounds and their properties. It can be concluded that, in the case of the **C1** and **C3** compounds, the coordination took place through the nitrogen atom contained in the pyridine ring [47]. Compounds **C2** and **C4**, which had high activity, had a very similar coordination. Cadmium coordinates through the nitrogen atom of the five-membered ring. The results obtained confirmed that the conversion of the ligands into the respective metal complexes significantly improved their anticancer properties. All of them showed much stronger activity than that of etoposide, which is a component of therapy for glioblastoma, neuroblastoma, and lung cancer. The most promising Cd (II) complex, **C2**, inhibited the growth of neuroblastoma cells five times more than etoposide, with an IC_50_ equal to 22.46 μM (vs. 115.25 μM for etoposide). The antibacterial efficiency of the complexes against Gram-positive bacteria decreased in the following order: **C1** > **C2** > **C4** > **C3**. Notably, all tested Cd complexes revealed good activity against yeasts in comparison to their ligands. The obtained compounds showed very good antitumor properties, which will allow for future research on the mechanisms of their binding to a particular target or active site (enzyme, protein), as well as their potential use as cytostatic drugs.

## Figures and Tables

**Table 1:** Temperature ranges and mass losses of the thermal decomposition stages of the cadmium (II) coordination compounds.

	C1	C2	C3	C4
I stage	35–220 °C 3.7%	35–220 °C 4.1%	35–180 °C 7.3%	35–185 °C 8.6%
II stage	430–760 °C 88.8%	360–475 °C 26.8%	265–465 °C 27.6%	320–453 °C 14.6%
III stage		475–705 °C 58.5%	465–715 °C 60.2%	460–775 °C 76.8%
Residual mass	7.5%	10.6%	4.9%	-

**Table 2:** Cytotoxic effects of the investigated compounds against glioblastoma (T98G), neuroblastoma, and lung carcinoma cell lines and normal human fibroblasts (CCD-1059Sk) determined with an MTT assay after 24 h of incubation.

IC_50_ ± SD [µM]				
Complex	T98G	SK-N-AS	A549	CCD-1059-Sk
**L1**	210 ± 12	>509	>509	>509
**C1**	Not determined due to low solubility in culture medium			
**L2**	178.3 ± 7.3	415 ± 17	220 ± 11	>509
**C2**	65.25 ± 0.63	22.5 ± 2.3	34.13 ± 0.55	44.07 ± 0.33
**L3**	>509	>509	>509	>509
**C3**	55.7 ± 2.5	34.53 ± 0.41	47.2 ± 2.1	40.38 ± 0.41
**L4**	>362	>362	>362	>362
**C4**	42.53 ± 0.96	25.3 ± 1.0	37.3 ± 2.1	27.66 ± 0.90
**Etoposide**	>170	115.3 ± 3.5	>170	>170

**Table 3:** In vitro antimicrobial activity of the tested ligands and complexes.

	Chemicals	L1	C1	L2	C2	L3	C3	L4	C4	Ref Drugs
Microorganism		MIC (mg/L)								
**Gram-positive bacteria**										**Van**
*S. aureus* ATCC 25923		>1000	500	1000	1000	>1000	1000	>1000	1000	0.98
*S. epidermidis* ATCC 12228		>1000	250	1000	500	>1000	1000	1000	250	0.98
*M. luteus* ATCC 10240		500	7.8	250	7.8	500	7.8	>1000	7.8	0.12
*E. faecalis* ATCC 29212		>1000	125	1000	125	>1000	>1000	1000	250	1.95
*B. subtilis* ATCC 6633		>1000	7.8	500	0.98	1000	7.8	1000	7.8	0.24
*B. cereus* ATCC 10876		>1000	500	250	500	1000	>1000	1000	250	0.98
**Gram-negative bacteria**										**Cip**
*S. typhimurium* ATCC 14028		>1000	1000	1000	>1000	>1000	125	>1000	1000	0.061
*E. coli* ATCC 25922		>1000	1000	1000	1000	>1000	125	>1000	500	0.015
*P. mirabilis* ATCC 12453		>1000	1000	1000	>1000	>1000	31.3	>1000	1000	0.03
*K. pneumoniae* ATCC 13883		>1000	1000	>1000	>1000	>1000	250	>1000	1000	0.12
*P. aeruginosa* ATCC 9027		>1000	>1000	>1000	>1000	>1000	125	>1000	>1000	0.49
**Yeasts**										**Nys**
*C. glabrata* ATCC 2091		>1000	62.5	>1000	31.3	>1000	31.3	>1000	31.3	0.48
*C. albicans* ATCC 102231		>1000	500	1000	125	>1000	125	500	125	0.24
*C. parapsilosis* ATCC 22019		>1000	15.6	>1000	7.8	1000	15.6	1000	7.8	0.24

## Data Availability

Not applicable.
